# A study on the anti-tumor mechanism of total flavonoids from Radix *Tetrastigmae* against additional cell line based on COX-2-mediated Wnt/β-catenin signaling pathway

**DOI:** 10.18632/oncotarget.16876

**Published:** 2017-04-06

**Authors:** Li Qinglin, Wenxiu Xin, Like Zhong, Luo Fang, Gang Cao, Ping Huang

**Affiliations:** ^1^ Zhejiang Cancer Hospital, Hangzhou, Zhejiang 310022, China; ^2^ Zhejiang Chinese Medical University, Hangzhou, Zhejiang 310053, China

**Keywords:** COX-2-Wnt/β-catenin, hepatocellular carcinoma, HCC, *Tetrastigmae*, TF

## Abstract

This study is to explore the effect of total flavonoids from *Radix Tetrastigmae* (TF) against hepatic cancer and discuss the acting mechanism. Proliferation of HepG2 cells was promoted by PGE2 and Butaprost. Using AH6809 as the positive control, the inhibitory effect of TF on additional cell line was detected through a CCK-8 assay, the apoptosis rate was detected by flow cytometry, and the nuclear morphology of cells were observed by Hochest33258 staining. Then PCR was applied to determine the mRNA expression. The corresponding Protein expression were determined by Western Blot. The effects of TF on the body weight, tumor growth volume and tumor inhibition rate were observed in nude mice model of human hepatocellular carcinoma by vivo experiments. The results showed TF had an obvious inhibitory effect on HepG2 cells with a significant dose-dependent manner (*P*<0.01). Pyknosis was found under the fluorescence microscope after TF treatment for 24h by Hochest33258 staining. Typical features of apoptosis were observed in HepG2 cells treated with TF and the apoptosis rate increased with increase of concentration of the TF. mRNA expression levels of GSK-3β, Akt, VEGF, COX-2 and β-catenin were down regulated greatly by the TF in HepG2 cells. Moderate and high concentrations of TF led to an obvious down regulation of GSK-3β, *p*-GSK-3β, Akt, *p*-Akt, VEGF, COX-2 and β-catenin in HepG2 cells, while *p*-β-catenin was up regulated. The tumor inhibition rates with high, medium and low dose of TF were 64.07%, 53.64%, 46.69%, respectively. The inhibitory effect of total flavonoids on tumor growth in mice was better than that of CTX, and the inhibitory effect of TF on tumor growth was less than CTX. TF exhibited a significant inhibitory effect on HepG2 cells, promoting the apoptosis of HepG2 cells in a dose-dependent manner. TF was also regulatory of the COX-2-Wnt/β-catenin signaling pathway, which was presumed to be the working mechanism of *Tetrastigmae*.

## INTRODUCTION

*Tetrastigmae* is the Radix from *Tetrastigma hemsleyanum*, which is endemic and rare traditional Chinese medicine plant and distributed in Zhejiang, Guangxi, Jiangxi, Fujian and other provinces. *Tetrastigmae* has obvious curative effect in promoting blood circulation and relieving pain function. In 1990s, the Chinese scholars have separated the flavonoids from the *Tetrastigmae*, and pharmacological tests showed that it has a good anti-tumor effect. *Tetrastigmae* contains quercetin, kaempferol, kaempferol glycoside-3-O-neohesperidin and other flavonoids [1]. As a popular medicine, *Tetrastigmae* was commonly used in tumor therapy in china. [2, 3]. Many studies have shown that *Tetrastigma* had a certain inhibitory effect on the growth and apoptosis of many tumor cell lines, and the anti-tumor effect was dose dependent *in vitro*.

Jiang Yuexian et al [4] studied the *Tetrastigmae* by Pharmacological experiment, and the research results showed that the *Tetrastigmae* had anti tumor effect and no mutagenicity. Ding Gangqiang et al [5] found that the ethyl acetate part of the *Tetrastigmae* extract had a strong inhibitory effect on the HepG2 activity of human hepatocarcinoma cells. Wang Zhen et al [6] studied the effect of the extract of *Tetrastigmae* on the apoptosis of human colon cancer cell line RKO, it was found that the extracts had anti proliferation and apoptosis inducing effect on RKO cells. Ni Kefeng et al [7] studied the inhibitory effect and mechanism of *Tetrastigmae* on tumor mice. The effect of the extract from *Tetrastigmae* on the immune function of mice was observed from the cellular immunity, humoral immunity, mononuclear macrophage phagocytosis and natural killer cells, by Xu Caiju et al [8]. The results showed that the extracts of *Tetrastigmae* had the function of enhancing the immunity of mice, and it was one of the mechanisms of anti tumor action. In summary, the antitumor effect of the *Tetrastigmae* can be summarized as two points, which can promote the apoptosis of cancer cells, and improve the cellular immunity and humoral immunity of the human body, especially it has obvious inhibitory effect on cancer, lung cancer, leukemia.

Our research group preliminary study found that the total flavonoids from *Radix Tetrastigmae* (TF) has a better anti HCC effect, can inhibit the release of inflammatory factors and cancer cytokines, compared with other compounds, TF advantage is very prominent. The safety of *Tetrastigmae* has been verified for a long time, and the content of TF in the original plant is relatively high, the extraction process is mature, these characteristics and advantages make the research more characteristic and practical significance. However, the study on the anti hepatocellular carcinoma(HCC) mechanism of *Tetrastigmae* was less. This study aimed to reveal the working mechanism of TF against HepG2 cells by focusing on the COX-2-mediated Wnt/β-catenin signaling pathway, providing clinical data for the development of novel drugs against HCC.

PGE2 (prostaglandin E2) is the product of arachidonic acid metabolism catalyzed by COX-2. PGE2 works through four specific G protein-coupled receptors (EP1, EP2, EP3 and EP4), promoting the proliferation, angiogenesis, invasion and metastasis of tumor cells while inhibiting tumor cell apoptosis. In this study, Butaprost was used to activate EP2, thus enhancing the proliferation of HepG2 cells treated by PGE2. AH6809, as an antagonist of EP2, inhibited the proliferation of HepG2 cells treated by PGE2. In this study, AH6809 was added as the positive control in the inhibition test of TF. Meanwhile, Butaprost was added to induce the proliferation of HepG2 cells and this effect was tentatively counteracted by TF.

## RESULTS

### Detection of cell proliferation by CCK-8 assay

#### Determining of concentrations of TF

After TF treatment for 48h, CCK-8 assay was applied and IC50 of TF was determined. Compared with the blank control group, all concentrations of TF had an inhibitory effect on HepG2 cells and the degree of inhibition increased with dose significantly (P<0.05). At 48h, IC50 of TF was 3.247mg/ml and the high, moderate and low concentrations of TF were set as 5mg/ml, 1.25mg/ml and 0.3125mg/ml, respectively (Table 1).

**Table 1:** Inhibitory effects of different concentrations of TF on the growth of HepG2 cells (X±S)

	Group	OD±SD	Inhibitive rate
	Negative control	0.789±0.014	0
TF IC50=3.247 mg/ml	20mg/ml	0.138±0.034	82.53%
	10mg/ml	0.207±0.04	73.71%
	5mg/ml	0.324±0.034	58.88%
	2.5mg/ml	0.404±0.022	48.75%
	1.25mg/ml	0.595±0.011	24.59%
	0.625mg/ml	0.663±0.032	15.92%
	0.3125mg/ml	0.688±0.024	12.80%
	0.15625mg/ml	0.722±0.02	8.54%
	0.078125mg/ml	0.744±0.023	5.73%
	0.0390625mg/ml	0.758±0.013	3.90%
	0.01953125mg/ml	0.779±0.013	1.29%

Compared with control group: * *P*<0.05; Compared with control group: **P<0.01

#### Determining the concentration of AH6809

As the positive control, different concentrations of AH6809 were used. IC50 of AH6809 was calculated based on CCK-8 assay 48h later. Compared with the blank control group, all concentrations of AH6809 had an inhibitory effect on HepG2 cells, in a dose-dependent manner (P<0.05). IC50 of AH6809 was 35.111uM at 48h, which was taken as the concentration for subsequent experiment (Table 2).

**Table 2:** Inhibitory effect of AH6809 on HepG2 cell growth (X±S)

	Group	OD±SD	Inhibitive rate
	Negative control	0.928±0.04	0
AH6809 IC50=35.111uM	160uM	0.23±0.019	75.26%
	80uM	0.305±0.025	67.13%
	40uM	0.43±0.023	53.71%
	20uM	0.611±0.012	34.18%
	10uM	0.698±0.022	24.74%
	5uM	0.727±0.026	21.62%
	2.5uM	0.794±0.019	14.46%
	1.25uM	0.826±0.036	10.99%
	0.625uM	0.844±0.043	9.07%
	0.3125uM	0.872±0.028	6.01%
	0.15625uM	0.914±0.024	1.55%

Compared with control group: * *P*<0.05; Compared with control group: **P<0.01

#### Determining the concentration of PGE2 and butaprost

PGE2 and Butaprost were applied to promote the proliferation of HepG2 cells, before the inhibition test using TF. Different concentrations of two drugs were tested and according to CCK-8 assay 48h later, both two inducers promoted cell proliferation compared with the blank control group, in a dose-dependent manner. However, after reaching a certain threshold, the ability of promoting cell proliferation declined and the inhibitory effect even appeared. From Table 3, the concentration of PGE2 was determined as 10uM; from Table 4, the concentration of Butaprost was determined as 20uM.

**Table 3:** The proliferation of HepG2 cells induced by PGE2 (X±S)

	Group	OD±SD	Promotion rate
	Negative control	0.987±0.01	
PGE2	160uM	0.822±0.023	-16.74
	80uM	0.891±0.029	-9.75
	40uM	1.053±0.021	6.71
	20uM	1.146±0.042	16.09
	10uM	1.252±0.018	26.89
	5uM	1.222±0.032	23.83
	2.5uM	1.145±0.014	15.99
	1.25uM	1.123±0.031	13.80
	0.625uM	1.054±0.045	6.79
	0.3125uM	1.02±0.04	3.36
	0.15625uM	0.991±0.013	0.39

Compared with control group: * *P*<0.05; Compared with control group: **P<0.01

**Table 4:** The proliferation of HepG2 cells induced by Butaprost (X±S)

	Group	OD±SD	Promotion rate
	Negative control	0.913±0.037	0
Butaprost	160uM	0.742±0.033	-18.71
	80uM	0.823±0.031	-9.81
	40uM	1.026±0.025	12.33
	20uM	1.111±0.022	21.64
	10uM	1.049±0.022	14.92
	5uM	1.028±0.008	12.57
	2.5uM	0.99±0.011	8.39
	1.25uM	0.974±0.021	6.66
	0.625uM	0.951±0.024	4.12
	0.3125uM	0.946±0.013	3.66
	0.15625uM	0.931±0.038	1.95

Compared with control group: * *P*<0.05; Compared with control group: **P<0.01

### Evaluation of inhibitory effects of TF on HepG2 cells

To evaluate the inhibitory effect of TF, HepG2 cells were first added with 10uM PGE2 and 10uM butaprost, using 35.111uM AH6809 as the positive control. Results showed that (Table 5), compared with the PGE2 control group, both three concentrations of TF had an obvious inhibitory effect on the cancer cells and the inhibition was enhanced with increasing dose (*P*<0.01). Compared with PGE2+AH6809 group, moderate and high concentrations of TF were much more inhibitory than AH6809 (*P*<0.05); low concentration of TF led to a weaker inhibition on HepG2 cells than AH6809, but the difference was of no significance. Compared with PGE2+Butaprost group, high concentration of TF had a significantly greater inhibition on HepG2 cells (*P*<0.05).

**Table 5:** Inhibitory effect of TF on the proliferation of HepG2 cells (X±S)

Group	OD±SD	Inhibitive rate
Negative control	0.886±0.009	0
PGE2	1.095±0.01	-23.63%
PGE2+TF (L)	0.771±0.02**	13.02%
PGE2+TF (M)	0.731±0.018**Δ	17.47%
PGE2+TF(H)	0.687±0.01**Δ	22.42%
PGE2+AH6809	0.758±0.013	14.40%
PGE2+Butaprost	1.115±0.051	-25.82%
PGE2+Butaprost+TF(H)	0.752±0.02^##^	15.10%

Compared with PGE2 group: * *P*<0.05; Compared with PGE2 group: **P<0.01

Compared with PGE2+AH6809 group: Δ*P*<0.05; Compared with PGE2+AH6809 group: ΔΔ*P*<0.01

Compared with PGE2+Butaprost group: ^#^*P*<0.05; Compared with PGE2+Butaprost group: ^##^*P*<0.01

### Detection of cell apoptosis by flow cytometry

**Figure 1:**
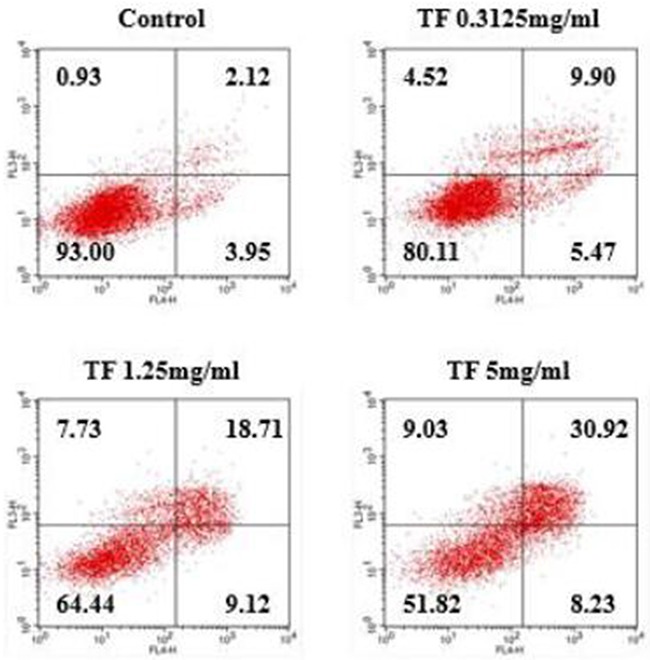
Study on the correlation between TF concentration and apoptosis of HepG2 cells

Following Annexin V-FITC/PI double staining, flow cytometry technique was applied to detect the cell apoptosis rate. According to Figure 1, TF treatment for 24h successfully induced apoptosis of HepG2 cells along with necrosis. The apoptosis rate was 6.07% in the blank control group, and that of low, moderate and high concentrations of TF was 15.37%, 27.83%, and 39.15%, respectively. As TF concentration increased, the apoptosis rate increased as well.

**Figure 2:**
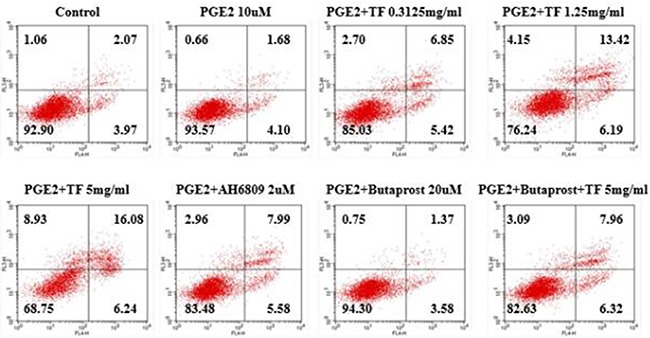
Effect of TF on apoptosis of HepG2 cells by flow cytometry

**Figure 3:**
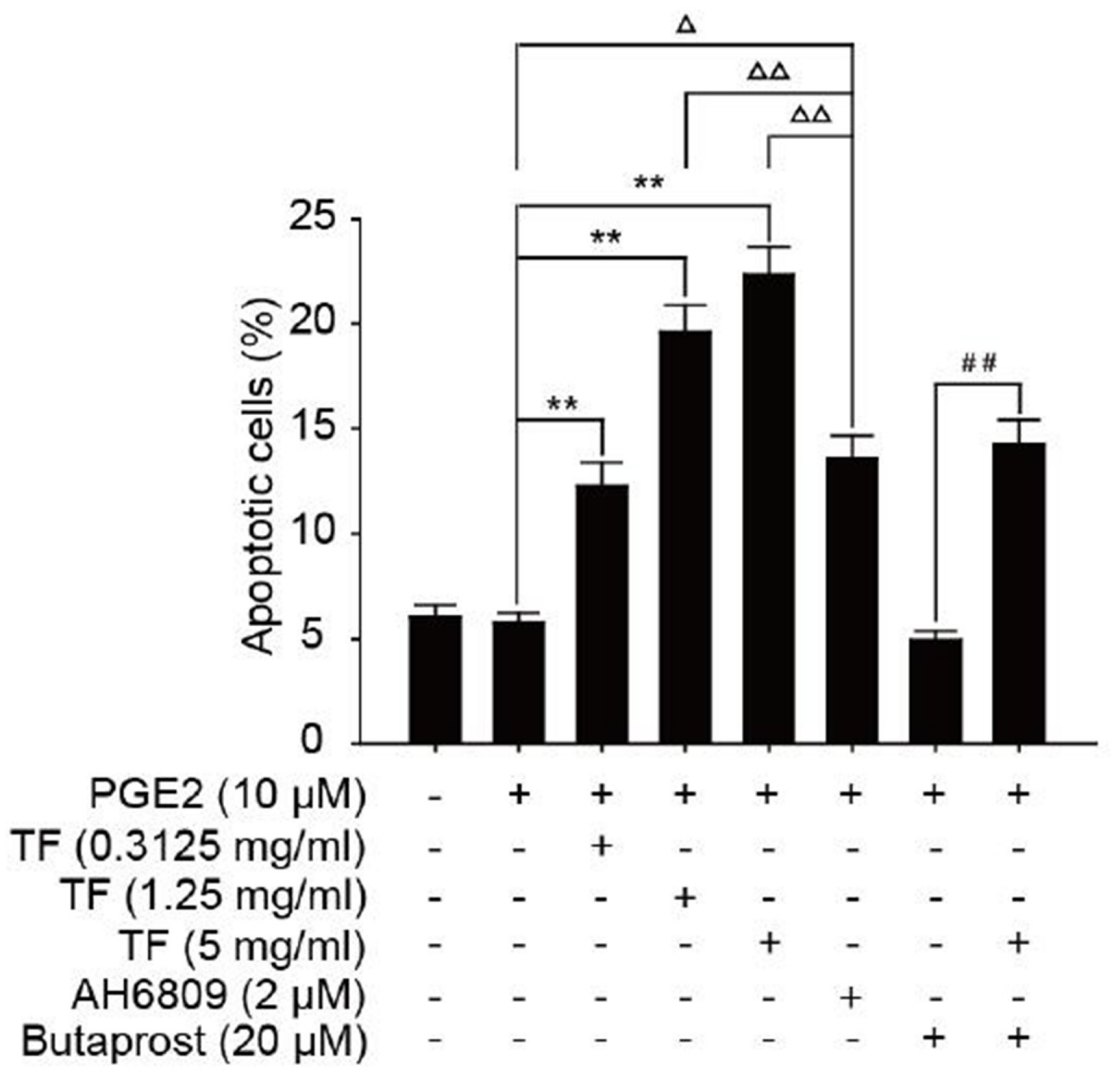
Effect of TF on apoptosis of HepG2 cells by flow cytometry (X±S) Compared with PGE2 group: * *P*<0.05; Compared with PGE2 group: **P<0.01. Compared with PGE2+AH6809 group: ^Δ^*P*<0.05; Compared with PGE2+AH6809group: ^ΔΔ^*P*<0.01. Compared with PGE2+Butaprost group: ^#^*P*<0.05; Compared with PGE2+Butaprost group: ^##^*P*<0.01.

Compared with PGE2 control group, all three concentrations of TF had a considerably higher apoptosis rate and the inhibition increased with dose (*P*<0.01). Compared with PGE2+AH6809 group, moderate and high concentrations of TF showed a significantly greater inhibition on HepG2 cells (*P*<0.01). However, low concentration of TF had a significantly weaker inhibition than AH6809 (*P*<0.05). Compared with PGE2+Butaprost group, high concentration of TF resulted in a marked increase of the apoptotis rate, which was 14.28% (*P*<0.01). However, the apoptosis rate of PGE2+Butaprost+TF group (14.28%) was lower than that of PGE2+TF group (22.32%). See Figure 2 and Figure 3.

### Observation of the effect of TF on morphology of HepG2 cells by Hoechst33258 staining

**Figure 4:**
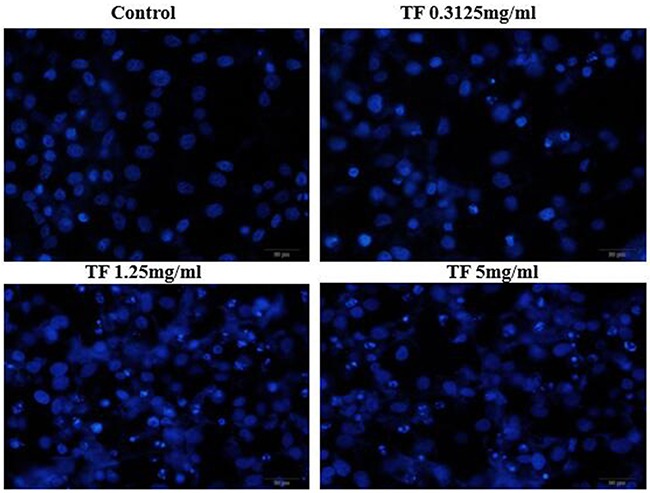
Effect of TF on morphology of HepG2 cells by Hoechst33258 staining (200X)

After treatments with different concentrations of TF (5mg/ml, 1.25mg/m and 0.3125mg/ml), Hochest33258 staining was performed and changes of nuclear morphology of HepG2 cells were observed under the fluorescence microscope. The results are shown in Figure 4. Normal cells had intact nuclei which were uniformly stained blue. After TF treatments for 24h, cells were pyknotic and showed typical features of apoptosis: nuclear shrinkage, fragmentation, and deeply stained. Apoptotic cells were densely and deeply stained, with higher brightness of nuclei. Under high concentration of TF, apoptotic bodies appeared and the percentage of apoptotic cells increased with TF concentration. This indicated that TF could induce the apoptosis of HepG2 cells.

### Detection of genes related to COX-2-Wnt/β-catenin pathway by PCR

**Figure 5:**
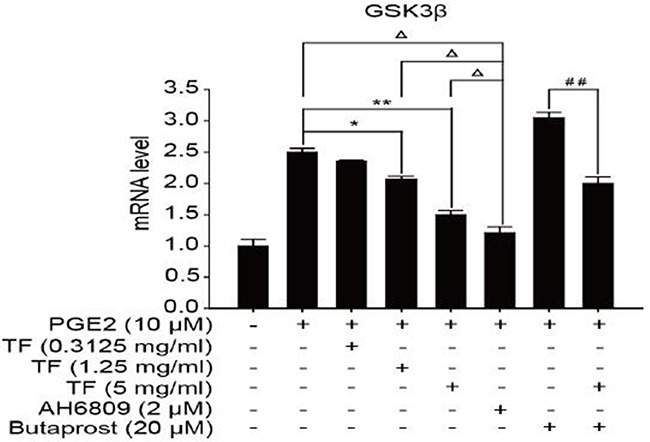
The expression of GSK-3β gene in different HepG2 cells groups

**Figure 6:**
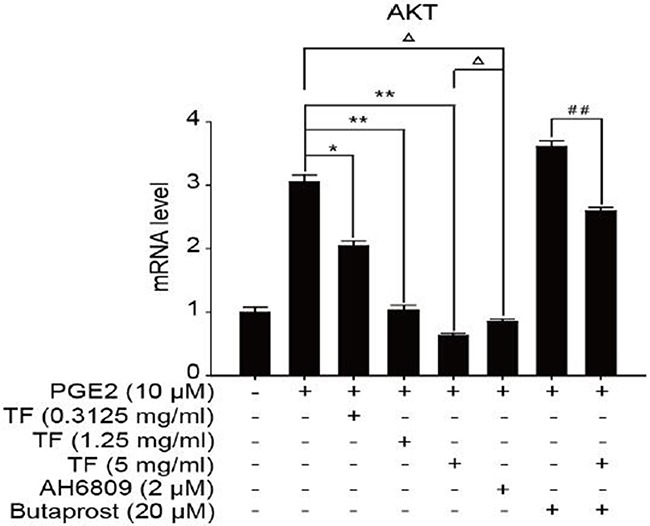
The expression of Akt gene in different HepG2 cells groups

**Figure 7:**
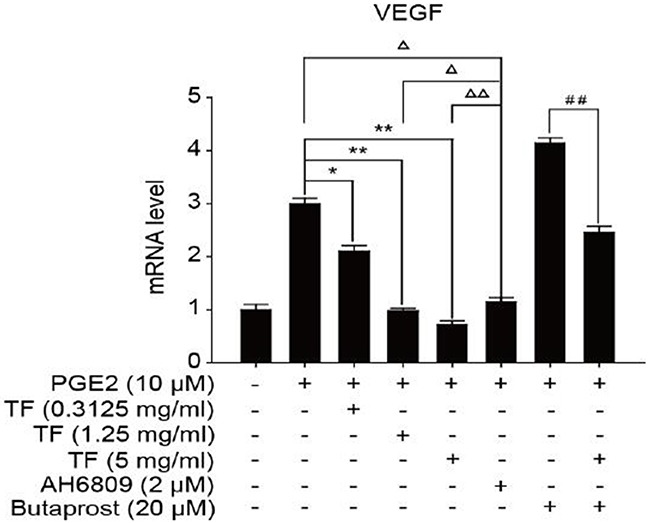
The expression of VEGF gene in different HepG2 cells groups

**Figure 8:**
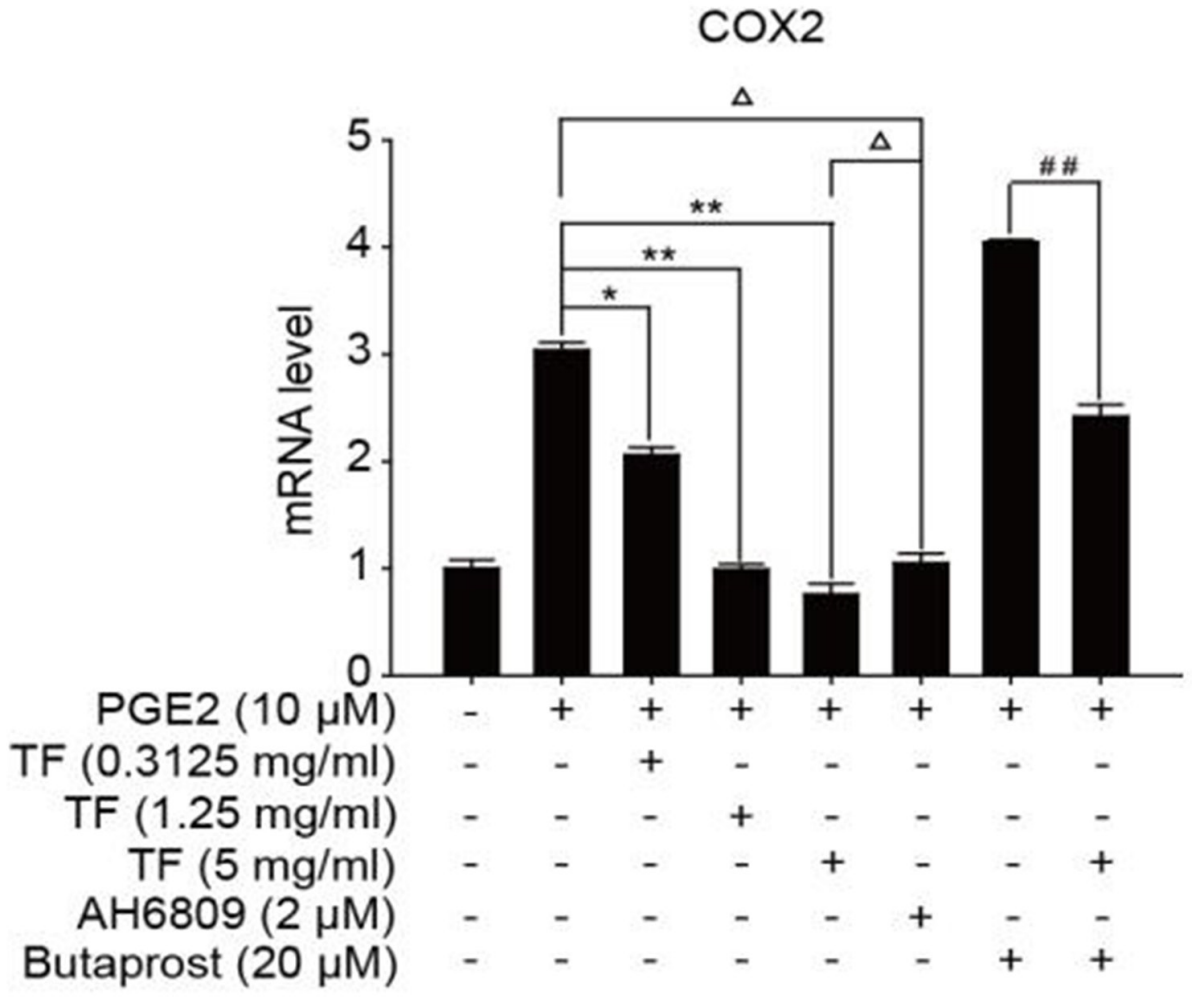
The expression of COX-2 gene in different HepG2 cells groups

**Figure 9:**
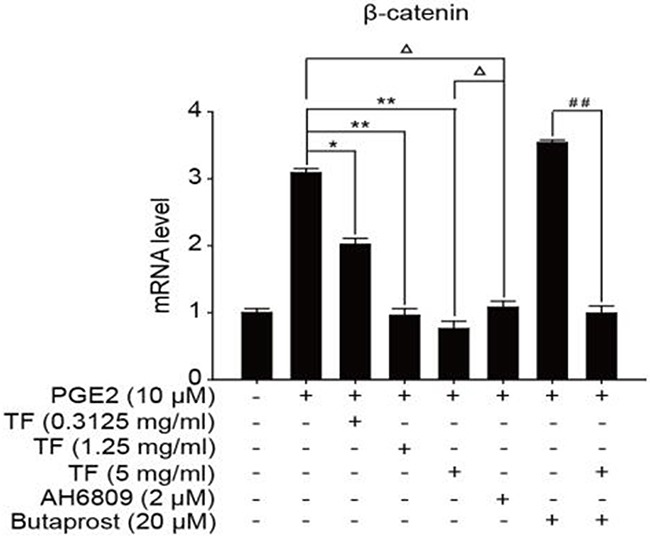
The expression of β-catenin gene in different HepG2 cells groups

To discuss the working mechanism of the anti-tumor activity of TF, PCR was applied to determine mRNA expression of GSK-3β, Akt, VEGF, COX-2 and β-catenin. Compared with the blank control group, both PGE2 and PGE2+Butaprost treatments upregulated mRNA expression levels of GSK-3β, Akt, VEGF, COX-2 and β-catenin in HepG2 cells to a great extent, especially for the PGE2+Butaprost treatment. Compared with PGE2 control group, all three concentrations of TF led to a considerable downregulation of GSK-3β, Akt, VEGF, COX-2 and β-catenin mRNA expression levels. This effect became more significant with higher concentration. Compared with PGE2+AH6809 group, high concentration of TF greatly downregulated mRNA expression levels of GSK-3β, Akt, VEGF, COX-2 and β-catenin in HepG2 cells. Under moderate concentration of TF, mRNA expression levels of COX-2, VEGF and β-catenin decreased significantly. Therefore, high concentration of TF was more potent in the regulation of COX-2-Wnt/β-catenin pathway in HepG2 cells than AH6809 (*P*<0.01 or *P*<0.05). But under low and moderate concentrations of TF, the regulatory effect was less potent than AH6809. Compared with PGE2+Butaprost group, high concentration of TF induced a significant downregulation of all genes related to COX-2-Wnt/β-catenin pathway (*P*<0.01) (Table 6). The effects of different treatments on the expression of each gene are presented in Figures 5, 6, 7, 8 and 9, respectively.

**Table 6:** Detection of genes related to COX-2-Wnt/β-catenin pathway by PCR (X±S)

Group	GSK3β	AKT	VEGF	COX2	β-catenin
Control	1.00±0.10	1.00±0.08	1.00±0.10	1.00±0.08	1.00±0.06
PGE2	2.50±0.06	3.05±0.11	3.00±0.10	3.04±0.07	3.09±0.06
PGE2+TF(L)	2.35±0.02Δ	2.04±0.08Δ*	2.10±0.11Δ*	2.06±0.07Δ*	2.02±0.09Δ*
PGE2+TF(M)	2.06±0.05Δ*	1.03±0.08**	0.98±0.05Δ**	0.99±0.05**	0.96±0.10**
PGE2+TF(H)	1.50±0.06Δ**	0.63±0.04Δ**	0.72±0.07ΔΔ**	0.76±0.10Δ**	0.76±0.11Δ**
PGE2+AH6809	1.21±0.09	0.85±0.04	1.15±0.08	1.05±0.09	1.08±0.09
PGE2+Butaprost	3.05±0.08	3.61±0.09	4.14±0.10	4.05±0.02	3.54±0.04
PGE2+Butaprost+TF(H)	2.00±0.10^##^	2.59±0.06^##^	2.46±0.11^##^	2.42±0.11^##^	0.99±0.11^##^

Compared with PGE2 group: * *P*<0.05; Compared with PGE2 group: **P<0.01

Compared with PGE2+AH6809 group: Δ*P*<0.05; Compared with PGE2+AH6809 group: ΔΔ*P*<0.01

Compared with PGE2+Butaprost group: ^#^*P*<0.05; Compared with PGE2+Butaprost group: ^##^*P*<0.01

### Detection of protein expressions related to COX-2-Wnt/β-catenin pathway by western blot

**Figure 10:**
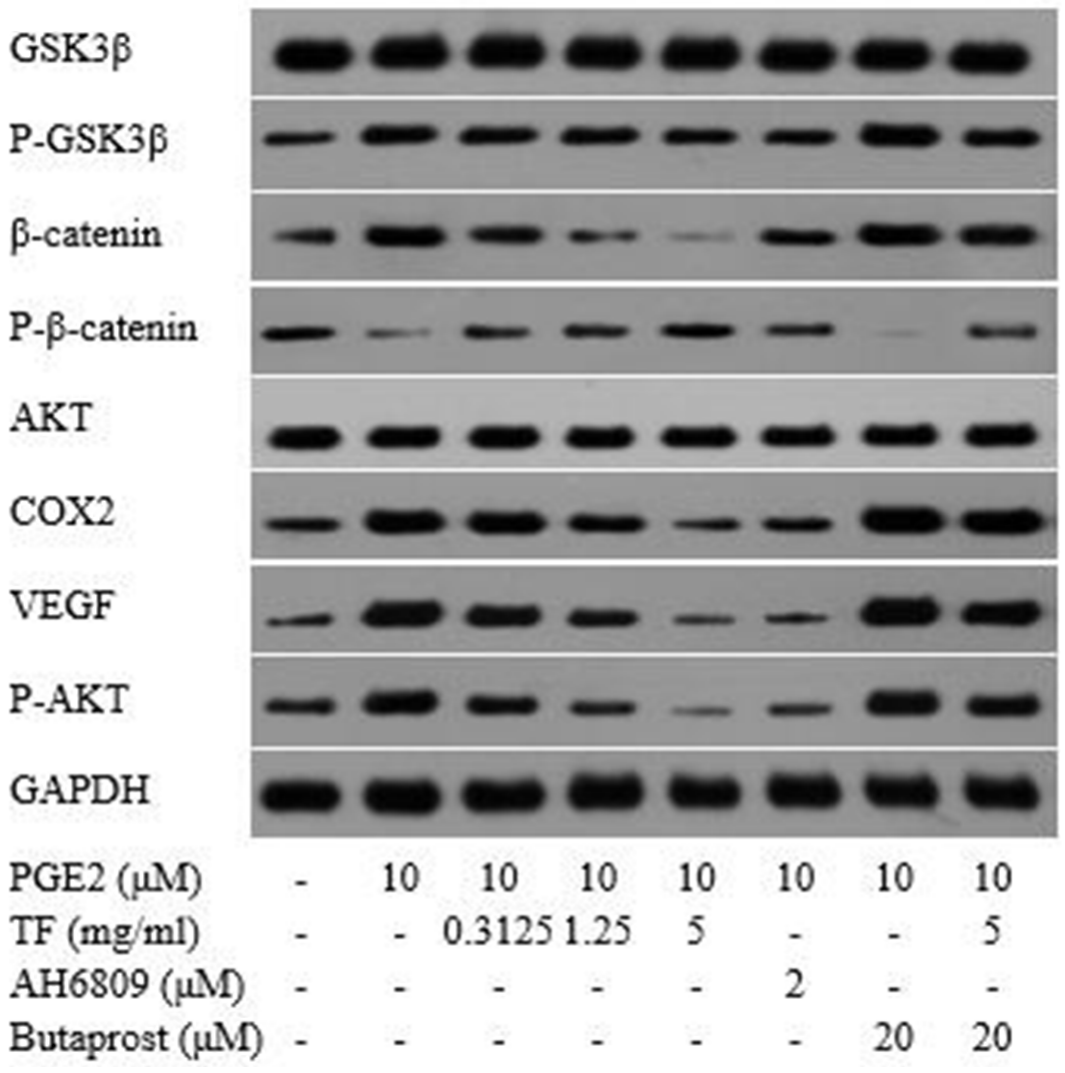
Detection of protein expressions related to COX-2-Wnt/β-catenin pathway by Western blot

Compared with the blank control group, both PGE2 and PGE2+Butaprost treatments led to a significant upregulation of *p*-GSK-3β, *p*-Akt, VEGF, COX-2 and β-catenin proteins, but a downregulatoin of *p*-β-catenin. The effect was more powerful with PGE2+Butaprost treatment. Compared with PGE2 control group, moderate and high concentrations of TF led to a significant downregulation of GSK-3β, *p*-GSK-3β, Akt, *p*-Akt, VEGF, COX-2 and β-catenin proteins in HepG2 cells; however, *p*-β-catenin was upregulated in a dose-dependent manner. Compared with PGE2+AH6809 treatment, high concentration of TF led to a considerable downregulation of *p*-GSK-3β, Akt, *p*-Akt, VEGF, COX-2 and β-catenin in HepG2 cells and an upregulation of *p*-β-catenin. From this it can be known that high concentration of TF was stronger than AH6809 in regulating the expression of proteins related to COX-2-Wnt/β-catenin pathway. In contrast, low and moderate concentrations of TF were less potent than AH6809. Compared with PGE2+Butaprost treatment, high concentration of TF significantly downregulated the expression of GSK-3β, *p*-GSK-3β, Akt, *p*-Akt, VEGF, COX-2 and β-catenin in HepG2 cells, wherease *p*-β-catenin was upregulated (as shown in Figure 10).

### Inhibitory effect of TF on tumor growth in mice with hepatocellular carcinoma *in vivo* experiments

**Figure 11:**
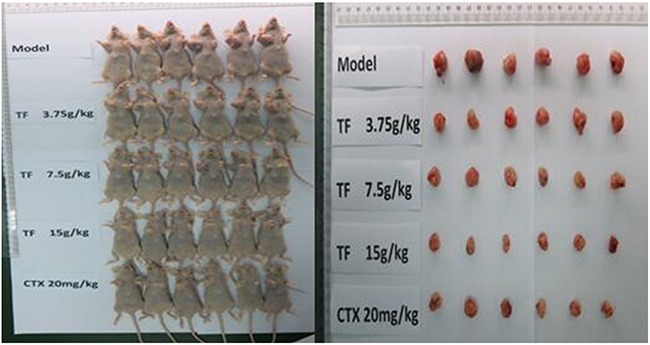
Inhibitory effect of TF on the growth of hepatocellular carcinoma in nude mice

At the end of the administration, we weighed and sacrificed the nude mice. The tumor was removed and weighed. From Table 7 and Figure 11, the inhibition rate of CTX was about 56.79%. The tumor inhibition rates of TF high, medium and low dose (15g/kg, 7.5g/kg, 3.75g/kg) were 64.07%, 53.64% and 46.69%. The inhibitory effect of TF high dose (15g/k) on tumor growth in mice was better than that of CTX, and the inhibitory effect of TF medium and low dose (7.5g/kg, 3.75g/kg) on tumor growth was less than CTX.

**Table 7:** Inhibitory effect of TF on the growth of hepatocellular carcinoma in nude mice (X±SD, n=6)

Animal group	Before administrationweight(g)	After administrationweight(g)	Tumor volume(cm^3^)	Tumor weight(g)	Inhibitionrate
NS	21.8±0.7	24.1±1.3	1.029±0.238	1.01±0.21	-
CTX	21.5±0.8	23.3±1.0	0.340±0.106**	0.44±0.12**	56.79%
TF-H	20.8±1.1	23.1±1.0	0.333±0.064**	0.36±0.09**	64.07%
TF-M	21.2±0.7	23.0±0.6	0.389±0.100**	0.47±0.12**	53.64%
TF-L	21.0±1.1	22.8±0.8	0.501±0.232**	0.54±0.20**	46.69%

Compared with NS: *p<0.05; Compared with NS: **p<0.01

## DISCUSSION

### Determination of TF concentration

Total flavonoid from Radix *Tetrastigmae* was prepared by TCM Processing Center, Zhejiang Chinese Medical University. The main components of the extract of total flavonoids from Radix *Tetrastigmae* were as follows: Procyanidin, Catechin, Procyanidin B2, Rutin, Isoquercitrin, Kaempferol-3-O-rutinoside, Astragalin, Quercitrin, Quercetin, Kaempferol. The purity of extract of total flavonoid was 62%. The purity and extraction rate of TF conformed to relevant standards. HepG2 cells were co-cultured with TF-containing serum. We used different concentrations of TF to interfere with HepG2 cells, and calculated the IC50 of the drug by CCK-8. We determined the high, medium and low doses of TF by IC50 and cell sensitivity to drugs, and the high, medium and low concentrations of TF were set as 5mg/ml, 1.25mg/ml and 0.3125mg/ml, respectively.

### The role of COX-2 mediated Wnt/β-catenin signaling pathway in HCC

Wnt/β-catenin signaling pathway plays an important role in regulating growth, movement and differentiation of cells and also in embryonic development, oncogenesis, tumor invasion and metastasis. This pathway has been recognized as a key signaling pathway in tumors [9, 10]. Exhibiting high degree of evolutionary conservativeness, Wnt/β-catenin pathway mainly consists of wnt proteins and glycogen synthase kinase-3β (GSK-3β). A large amount of unphosphorylated β-catenin in the cytoplasm enters the nuclei through transposition, binding to TCF/LEF factors and generating transcription complexes that activate the transcription of target genes, such as VEGF [11]. These transcriptional products play a key role in cell proliferation, apoptosis inhibition, tumor invasion and angiogenesis. However, in normal cells, Wnt/β-catenin pathway is usually quiescent, where β-catenin is phosphorylated by GSK-3β and then degraded via the ubiquitin-dependent pathway. But in some tumor cells where one or several components of Wnt/β-catenin pathway are abnormal, β-catenin is not phosphorylated and degraded. As a result, β-catenin accumulates in the nuclei, which activates the transcription of the target gene. Study shows that β-catenin signaling pathway is activated in HCC [12]. In another study, β-catenin accumulates in the nuclei of HCC cells with the upregulation of COX-2, which is the target gene regulated by β-catenin [13]. That means β-catenin accumulation can be prevented by inhibiting COX-2 expression in HCC, which blocks the Wnt/β-catenin signaling pathway and downregulates VEGF. Therefore, looking for an antagonist of COX-2-Wnt/β-catenin pathway may provide a treatment for HCC. With this goal in mind, we detected mRNA and protein expression levels of GSK-3β, Akt, VEGF, COX-2 and β-catenin related to COX-2-Wnt/β-catenin signaling pathway in HepG2 cells by PCR and Western Blot, respectively. Based on the detection results, the inhibitory mechanism of TF on HepG2 cells was discussed through the regulation of COX-2-Wnt/β-catenin pathway.

### Research design and primary outcome

The purpose of this study is to confirm and evaluate the inhibitory effect of TF on proliferation of hepatocellular carcinoma cells, we used AH6809 inhibitors as the positive control group, induced by PGE2 and Butaprost activator to promote proliferation of hepatocellular carcinoma cells, to highlight the inhibitory effect of TF on proliferation of hepatocellular carcinoma cells. Prostaglandin E2 (PGE2) is four arachidonic acid metabolites by COX-2 catalysis, through four kinds of specific membrane G protein coupled receptors (EP1, EP2, EP3 and EP4) play a role, it can promote tumor cell proliferation, angiogenesis and metastasis, can inhibit the apoptosis of tumor cells. Butaprost is an activator of EP2 receptor, which can enhance the proliferation of HepG2 cells by promoting PGE2. AH6809 is a EP2 receptor antagonist, which can inhibit the proliferation of HepG2 cells by PGE2.

In this study, the inhibitory effect of TF on HepG2 cells was detected through a CCK-8 assay. The apoptosis rate of HepG2 cells was detected by flow cytometry. Following Hochest33258 staining, changes of nuclear morphology of cancer cells were observed under the fluorescence microscope. In order to clarify the role of TF in regulating the proliferation and promoting apoptosis of HCC cells through the regulation of COX-2-Wnt/β-catenin signaling pathway, PCR was applied to determine the mRNA expression of GSK-3β, Akt, VEGF, COX-2 and β-catenin, and protein expression levels of GSK-3β, p-GSK-3β, Akt, p-Akt, VEGF, COX-2, β-catenin and p-β-catenin were determined by Western Blot. The effects of total flavonoids on the body weight, tumor growth volume and tumor inhibition rate were observed in nude mice model of human hepatocellular carcinoma by vivo experiments. The results showed that TF exhibited a significant inhibitory effect on HepG2 cells, promoting the apoptosis of HepG2 cells in a dose-dependent mannner. TF was also regulatory of the COX-2-Wnt/β-catenin signaling pathway, which was presumed to be the working mechanism of TF.

We have not only done the HepG2 cell line, but also made other hepatoma cell lines(HHCC,SMMC-7721). The results were in agreement with the HepG2 cell line, and we also did the study of vivo experiments in the nude mice model of liver cancer. Taking into account the length of the content of the article, we did not write these contents.

### Comparison with other studies

There are abundant clinical basis in the research of prevention and treatment of liver cancer with *Tetrastigmae* in China. However, there is no experimental research on the pharmacodynamic material basis of *Tetrastigmae* against liver cancer. This study is the first to clarify the anti hepatoma effect and mechanism of total flavonoids from Radix Tetrastigmae. The results showed that the safety and efficacy of total flavonoids from Radix *Tetrastigmae*. This study reveal the working mechanism of TF against HepG2 cells by focusing on the COX-2-mediated Wnt/β-catenin signaling pathway, providing clinical data for the development of novel drugs against HCC. This study provided a new approach for the study of innovative drugs against liver cancer.

## MATERIALS AND METHODS

### Materials

#### Cell line and animal

Hepatocellular carcinoma cell lines (HepG2) were provided by Jiangsu Kaiji biological technology Co., Ltd. The complete culture medium of cell lines was 90%DMEM+10%FBS, which was cultured in the culture medium of 37°C and 5%CO_2_ saturated humidity.

BALB/c nude mice (Changzhou Vince Biotechnology Development Co., ltd. Certificate NO: 201510525). Animal production license: SCXK (Su 2011-0003), Experimental animal license: SYXK (Su) 2011-0036, Age: 4-5 week; weight: 18-22g, Gender: female, Animal number: 30.

#### Medicines and reagents

**Figure 12:**
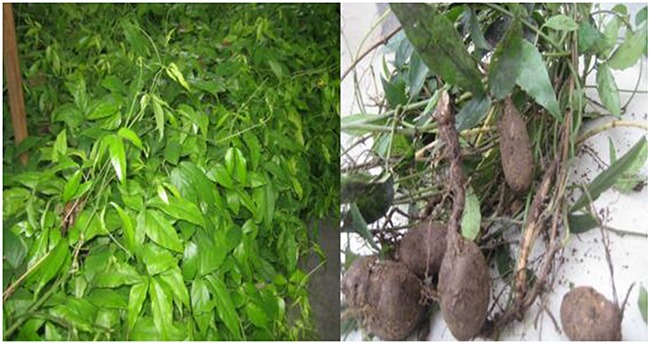
The *Tetrastigmae* Herba and *Tetrastigmae Radix*

The *Tetrastigmae* Herba was collected from Lishui (Figure 12), Zhejiang province, Voucher specimens were identified by Xi-lin Chen professor of Zhejiang Chinese Medicine University. Total flavonoids from Radix *Tetrastigmae* was prepared by the processing technology research center of Chinese medicine of Zhejiang Chinese Medicine University.

0.25% Tripsin-EDTA(Jiangsu Kaiji biological technology Co., Ltd., China, batch number: KGY001); Cell Counting Kit-8 (DOJINDO Laborataries Co., Ltd., Japan, batch number:CP736); DMEM (GIBCO Co., Ltd., USA, batch number:12800-0820);FBS(ExCell Biology Co., Ltd.,USA,batch number:FBS500); AnnexinV-APC/7-AAD (Jiangsu Kaiji biological technology Co., Ltd., China, batch number: KGA1024) Hoechst33258(Jiangsu Kaiji biological technology Co., Ltd., China, batch number: KGA211); TRIzol (Invitrogen Co., Ltd.,USA,batch number: 15596-026;Real time PCR Master Mix(SYBR Green) (TOYOBO,Japan); COX2 (Jiangsu Kaiji biological technology Co., Ltd., China, batch number: BA0738) β-catenin (Jiangsu Kaiji biological technology Co., Ltd., China, batch number: KG21520-2); *p*-β-catenin (Jiangsu Kaiji biological technology Co., Ltd., China, batch number: KG11116-2); GSK-3β (Jiangsu Kaiji biological technology Co.,Ltd., China, batch number: KG21002-2); *p*-GSK-3β (Jiangsu Kaiji biological technology Co., Ltd., China, batch number: KG11002-2); AKT (Jiangsu Kaiji biological technology Co., Ltd., China, batch number: KG21054-2); *p*-AKT (Jiangsu Kaiji biological technology Co., Ltd., China, batch number: KG11055-2);VEGF(Jiangsu Kaiji biological technology Co., Ltd., China, batch number: BA0407).

#### Instrument

Mettler Toledo AL104 analytical balance (Switzerland Mettler Toledo); BIOPAC mp150 type 16 channel physiological signal recording and analysis system (US BIOPAG); Delixi constant temperature operation (Delixi Group); AD113B08114 polarized light microscope (Olympus, Japan). Flow cytometry (FACS Calibur Becton-Dickinson, USA).5%CO2 incubator HEPA Class 100 was purchased from Thermo Fisher. Water bath termostat oscylator was bought from Taicang Laboratory Instrument Manufacture. CO2 incubator (5410-220) was made by Precision Scientific. Laminar flow hood was made by Suzhou Antai Air Technology Co., Ltd (BCM-1000A). Counter top centrifuge was purchased from Beijing Jingli Centrifuge Co., Ltd (LDZ 5-2). Electric Thermostat water bath (DK-450 B type) was purchased from Shanghai Senxin Experimental Instrument Co., Ltd. Liquid nitrogen tank was MVE CRYOSYSTEM750. Microplate reader was made by Thermo Fisher (3001-1249). UV spectrophotometer was made by Eppendorf. PCR gene amplifier was made by Bio-Rad Laboratories, Inc (US). iQ TM5 Real-Time quantitative PCR was made by Bio-Rad Laboratories, Inc (US).

### Methods

#### Preparation of TF

*Tetrastigmae*, weighing 5kg, was added with 6 times volume of 60% ethanol for heat reflux extraction. Extraction was performed for 3 times, 1.5 h each time. The filtrates were combined and concentrated under reduced pressure to obtain the extract. The extraction was added into the HPD826 macroporous adsorption resin column, and washed by the water and the different concentration of ethanol, and the corresponding elution liquid was collected, and the ethanol was recovered, concentrated into a thick paste, dried in vacuum. Finally, Total flavonoids from Radix Tetrastigmae were obtained. All analysis of extracts were performed using high performance liquid chromatography tandem mass spectrometry (HPLC-MS/MS) methods. Chromatographic separations were achieved using a Waters Acquity BEHC18 column (2.1mm×150 mm, particle size 1.7μm, Waters, Wexford, Ireland). The mobile phase conditions were as follows: A was water with 0.5% formic acid, and B was acetonitrile. The gradient elution procedure was that 0-10 min, A:B=90:10 (v/v); 10-15 min, A:B by 90:10 gradually rose to 70: 30 (v/v); 15-45 min, A:B by 70:30 gradually rose to 10:90 (v/v) 45-60min, A:B=10:90 (v/v).

#### Cell culture

Human hepatic cancer HepG2 cells were cultured in RPMI-164 medium containing 10% fetal bovine serum and placed at 37°C in a 5% CO2 incubator. The cells grew in monolayers, and the culture medium was discarded when the cells covered over 80% of the culture flask bottom. Trypsin (0.25%) was added to digest the cells for 1-2 min. When the cells became round, equal volume of serum-containing culture medium was added to terminate digestion. Cells were suspended by blowing with a pipette and transferred to a 15ml centrifuge tube for centrifugation at 1000r/min for 5min. With supernatant discarded, 1-2ml of culture medium was added. The cells were resuspended and transferred to a new culture flask. The cells were cryopreserved by adding 1ml of cryopreservation solution for every 5×10^6^ cells. Before use the cells were resuscitated and dethawed rapidly at 37°C. Cryopreservation solution was removed by centrifugation and culture medium was added. The cells adhered to the wall the next day and proliferated actively 2-3 days after inoculation. Cells reaching the log phase were harvested.

### *In vitro* cell experiment

#### Detection of proliferation of HepG2 cells treated with TF using CCK-8 assay

Different groups were set up, including TF treatment groups, inducer (PGE2, butaprost) group and AH6809 group, for determining the concentrations. Different concentrations of the above drugs were applied to the HepG2 cells and the cell proliferation was observed by the microscope after 48h. Low, moderate and high concentrations of TF were evaluated based on the degree of inhibition on HepG2 cells.

For drug interventions, the following groups were set up: PGE2, PGE2+TF (H), PGE2+TF (M), GE2+TF (L), PGE2+AH6809, PGE2+Butaprost and PGE2+Butaprost+TF(H). Meanwhile, negative control using only solvent was administered. HepG2 cells were grown on RPMI-164 medium supplemented with 10% FBS and inoculated to the 96-well plate at the density of 3000 cells per well for 24h, as the negative control. Then CCK-8 solution was added at a volume ratio of 1:10 72h after inoculation. The cells were cultured for 2.5h before measuring OD value at 450nm using a microplate reader. Cell proliferative activity was calculated as follows: proliferative activity. Taking the mean of the control group as 100%, the ratio of proliferative activity between other groups and the control group was the cell proliferation rate. Inhibition rate=1-proliferation rate.

#### Detection of the effect of TF on cell apoptosis by Annexin-V APC/7-AAD double staining

Log-phase cells were inoculated to the 6-well plate and the cells adhered to the wall the next day. Culture media containing different drugs were added into each group with the setting of negative control; After treatment for 48h, the cells were digested with 0.25% trypsin (free of EDTA); The cells were washed with PBS twice (centrifugation at 2000rpm for 5min) and 5×10^5^ cells were collected; Cells were suspended by adding 500μL of binding buffer; Cells were first added with 5μL Annexin V-APC and mixed well, and then with 5μL 7-AAD and mixed well; Cells were left at room temperature in the dark for 5-15min. Cell apoptosis was detected using a flow cytometer.

#### Hoechst33258 staining

Cell pretreatment: The cell specimens were air dried and fixed in 4% paraformaldehyde for 30min or overnight to improve cell permeability. Cells were washed with PBS for 3 times, 3min each time. The cells were soaked in PBS for 5 min and washed for 3 times. Cells were added with sufficient amount of Hoechst 33258 stain at room temperature for 10min. The cells were soaked in PBS for 5 min and washed for 3 times. The slides were sealed with anti-fluorescence quenching liquid and observed under the fluorescence microscope.

#### Detection of gene expression by QPCR

Total RNA was extracted and converted to cDNA using the RevertAidTM First Strand cDNA synthesis kit following the supplier's instructions. GAPDH was used as a control for adjusting the relative of total RNA. The thermal cycles were: 96°C for 30 s, 65°C for 30 s, and 72°C for 30 s for 30 cycles for CELF1, and 95°C for 30 s, 58°C for 30 s, and 72°C for 30 s for 30 cycles for GAPDH. The primers (Invitrogen, China) used for PCR were as follows: qhGAPDH primer (115bp): Sense primer: 5- CATCTTCTTTTGCGTCGCCA -3, Antisense primer: 5- TTAAAAGCAGCCCTGGTGACC -3; qhAKT primer(67bp):Sense primer 5-GCAGCACGTGTACGA GAAGA-3, Antisense primer: 5- GGTGTCAGTCTC CGACGTG-3; qhCOX2 primer (90bp): Sense primer: 5- ATGCTGACTATGGCTACAAAAGC-3,Antisense primer: 5-TCGGGCAATCATCAGGCAC-3, qhGSK3β primer (89bp):Sense primer: 5-AGACGCTCCCTGTGATTTATGT-3,Antisense primer:5- CCGATGGCAGATTCCAAAGG-3; qhVEGF primer (94bp):Sense primer:5- GCAGCTTGA GTTAAACGAACG-3, Antisense primer: 5-GGTTCCCG AAACCCTGAG-3; qhcatenin primer (197bp):Sense primer: 5-TGCCAAGTGGGTGGTATAGAGG-3,Antisense primer: 5- CAGTGGGATGGTGGGTGTAAGA -3. The PCR reactions for GSK-3β, Akt, VEGF, COX-2,β-catenin and GAPDH were analyzed by 2% agarose gel electrophoresis. Reverse transcription PCR was carried out at least three times.

#### Detection of protein expression by western blot

Total protein extraction: The cells were digested with trypsin and washed with precooled PBS for twice. Into each tube 200ul of precooled lysis buffer was added, mixed well and placed in an ice bath for 30min. Vortex oscillation was performed for 10s every 5min, followed by centrifugation at 13000×g and 4°C for 10min. Supernatant was collected, subpackaged and preserved at -20°C. OD values were measured at 520nm for the samples and the standards. The standard curves were plotted for each protein standard and the concentrations of proteins in each group were calculated. Transfer buffer was precooled at 4°C. After transfer, NC membranes were taken out, labeled and rinsed with TBST for three times, 10min each time. After reaction with the secondary antibodies, the secondary antibodies were recycled and the membranes were washed with TBST for 3 times, 5-10min each time. Band density was measured using G: BOX Chemi XR5 Imaging System, and the grayscale value was analyzed by Gel-Pro32 software.

### *In vivo* experiment

#### Establishment of nude mice model of hepatocellular carcinoma

HepG-2 cells was collected and cultured, and the concentration was 1×107 /ml. Then each nude mouse was inoculated with 0.1ml on the right axillary subcutaneous.

#### Grouping and administration

Model group (NS): normal saline, 0.1ml/10g, once a day

Cyclophosphamide (CTX): ij, 20mg/kg, 0.1ml/10g, once every 2 days

TF high dose group (TF-H): ig, 15g/kg, 0.1ml/10g, once a day

TF medium dose group (TF-M): ig, 7.5g/kg, 0.1ml/10g, once a day

TF low dose group (TF-L): ig, 3.75g/kg, 0.1ml/10g, once a day

We used vernier caliper to measure the diameter of tumor, when the tumor grew to 75-100 mm^3^, the animal were randomly divided into four groups mentioned above, 6 rats in each group. At the same time, nude mice were weighed and started to be administered. After 3 weeks of administration, the rats were weighed and were treated with surgical removal of tumor masses and weighing

#### Observation index

Ttumor volume (TV):

TV=1/2×a×b^2^a and b represent the length and width, respectively.

Tumor growth inhibition rate:

### Statistical analysis

All results are the mean± standard deviation (x¯ ±s) said, using SPSS20.0 version software for analysis of variance between groups were compared using t test, P<0.05 was considered significant difference, P<0.01 for the pole significant differences. Airway reactivity compared using repeated ANOVA, there was a significant difference between groups effect, the use of single-factor analysis of variance between dose groups analysis of variance. P<0.05 was considered significant difference, P <0.01 for a very significant difference.
